# PMSE Centennial: Celebration of Success and New Frontiers in Polymer Materials Science and Engineering

**DOI:** 10.1021/acsmacrolett.5c00626

**Published:** 2025-09-26

**Authors:** Melissa A. Grunlan, LaShanda T. J. Korley, Qinghuang Lin, Christopher L. Soles, Rigoberto Advincula, Arthi Jayaraman, Elizabeth Cosgriff-Hernandez, Elsa Reichmanis, Jodie L. Lutkenhaus, Rodney D. Priestley, Brigitte Voit, Thomas H. Epps

**Affiliations:** † Department of Biomedical Engineering, Department Materials Science and Engineering, and Department of Chemistry, 14736 Texas A&M University, College Station, Texas 77843, United States; ‡ Department of Materials Science and Engineering and Center for Research in Soft Matter and Polymers (CRiSP), University of Delaware, Newark, Delaware 19716, United States; § Canon Nanotechnologies, Austin, Texas 78758, United States; ∥ 596358 NIST Materials Measurement Laboratory, Gaithersburg, Maryland 20899, United States; ⊥ Center for Nanophase Materials Sciences, Oak Ridge National Laboratory, Oak Ridge, Tennessee 37996, United States; # Department of Chemical Engineering, University of Tennessee at Knoxville, Knoxville, Tennessee 37996, United States; ○ Department of Chemical and Biomolecular Engineering, Department of Materials Science and Engineering, and Data Science Institute, 5972 University of Delaware, Newark, Delaware 19716, United States; ● Department of Biomedical Engineering, 12330 The University of Texas at Austin, Austin, Texas 78712, United States; □ Department of Chemical and Biomolecular Engineering, 1687 Lehigh University, Bethlehem, Pennsylvania 18015, United States; ■ Artie McFerrin Department of Chemical Engineering, Department of Materials Science and Engineering, 14736 Texas A&M University, College Station, Texas 77843, United States; △ Department of Chemical and Biological Engineering, Princeton Materials Institute, 6740 Princeton University, Princeton, New Jersey 08544, United States; ▲ Organic Chemistry of Polymers, TUD Dresden University of Technology, Dresden 01062, Germany; ▼ Division Macromolecular Chemistry, Leibniz Institute of Polymer Research Dresden, Dresden 01069, Germany; ▽ Department of Chemical and Biomolecular Engineering, Center for Plastics Innovation (CPI), Department of Materials Science and Engineering, and Center for Research in Soft Matter and Polymers (CRiSP), 5972 University of Delaware, Newark, Delaware 19716, United States

## Abstract

In 2024, the American Chemical Society (ACS), Division of Polymeric Materials: Science and Engineering (PMSE), celebrated its centennial. This historic occasion was marked at the 2024 Spring ACS Meeting in New Orleans with a Centennial Symposium entitled “*PMSE Centennial: Celebration of Success and New Frontiers in Polymeric Materials Science and Engineering*”. The symposium reflected on past scientific breakthroughs, technological advancements, and new frontiers in the field of polymeric materials science and engineering. Eight thematic areas comprised the symposium: *Advanced Manufacturing*, *AI and Materials Discovery*, *Biomaterials*, *Electronic Materials*, *Energy*, *Entrepreneurship*, *Smart Materials*, *and Sustainability*. The 31 distinguished speakers, representing academia, industry, and national laboratories, shared their unique perspectives. Within this Viewpoint, distinguished speakers have expanded on the symposium’s themes, summarizing key takeaways, identifying critical challenges, and exploring opportunities for continued advancements in polymer science.

## Graphical abstract

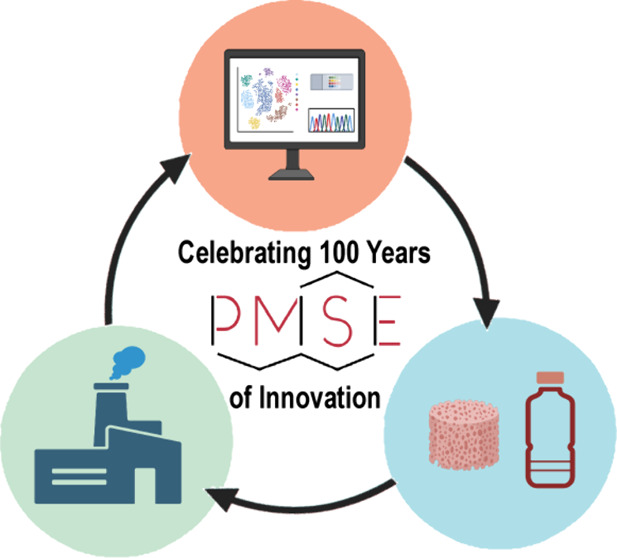

## Introduction - Grunlan, Korley, Lin, and Soles

In 2024, the American Chemical Society (ACS), Division of Polymeric Materials: Science and Engineering (PMSE), marked a historic milestone, celebrating its 100th anniversary.^1^ The division’s name has evolved since its inception in 1923 as the Division of Paint, Varnish, and Plastics, to the Division of Paint, Varnish and Plastics to the Division of Paint, Plastics and Printing Ink Chemistry (PPPIC) in 1952, to the Division of Organic Coatings and Plastic Chemistry (OCPC) in 1961, and ultimately to the Division of PMSE in 1986. Today, PMSE is a premier global scientific society dedicated to advancing the field of polymeric materials science and engineering. With more than 3000 members, it is one of the largest polymer-focused professional societies in the world today. PMSE’s mission is to foster a global community of scientists and engineers advancing polymeric materials through science and engineering. In this endeavor, PMSE is grateful for the synergistic relationship with the Division of Polymer Chemistry (POLY).

Over the past 100+ years, scientific breakthroughs, technological advancements, and major industry products have improved the living standards of billions of global citizens. The following is a nonexclusive list of some of these major milestones.

**1839**: Charles Goodyear discovered vulcanization, a process that strengthens natural rubber by heating it with sulfur, leading to more durable rubber products.

**1907**: Leo Baekeland developed Bakelite, the first fully synthetic polymer, which is nonconductive, heat-resistant, and moldable, widely used in electrical insulators and automotive parts.

**1920**: Hermann Staudinger proposed the macromolecular theory, suggesting that polymers are long chains of covalently bonded repeating units, laying the foundation for the field of modern polymer science.

**1923**: The ACS established the Division of Paint, Varnish, and Plastics, reflecting the growing importance of polymeric materials in science and industry.

**1935**: Wallace Carothers developed nylon, the first synthetic fiber, revolutionizing the textile industry with its strength and elasticity.

**1938**: Roy Plunkett discovered polytetrafluoroethylene (PTFE), later branded as Teflon, known for its nonstick properties and chemical resistance.

**1939**: Polyethylene was first synthesized, becoming one of the most widely used plastics due to its versatility and low cost.

**1941**: Polyethylene terephthalate (PET) was developed, leading to the production of polyester fibers and later becoming popular for beverage bottles.

**1942**: Styrene–butadiene rubber (SBR) was produced on a large-scale during World War II as a synthetic alternative to natural rubber.

**1946**: Herman Mark established the Polymer Research Institute at Brooklyn Polytechnic, the first U.S. research facility dedicated to polymer research.

**1953**: Hermann Staudinger was awarded the Nobel Prize in Chemistry for his contributions to the understanding of macromolecular chemistry.

**1954**: Giulio Natta and Karl Ziegler developed catalysts for the polymerization of alkenes, leading to the production of isotactic polypropylene.

**1958**: Kevlar, a high-strength aramid fiber, was invented by Stephanie Kwolek, later used in bulletproof vests and other applications requiring high tensile strength.

**1963**: Karl Ziegler and Giulio Natta were awarded the Nobel Prize in Chemistry for their work on the polymerization of alkenes.

**1965**: Polycarbonate was introduced, known for its high impact resistance and optical clarity, used in applications like eyewear lenses and CDs.

**1974**: Paul Flory was awarded the Nobel Prize in Chemistry for his work on the physical chemistry of macromolecules.

**1980**: Conductive polymers are discovered, leading to developments in organic electronics, including organic light-emitting diodes (OLED)­s.

**1986**: The ACS Division of Polymeric Materials: Science and Engineering (PMSE) adopts its current name, reflecting the broadening scope of the field.

**1987**: Grant Willson, Jean Fréchet, and Hiroshi Ito invented chemically amplified photoresists that have enabled the manufacturing of modern microelectronic products from mobile phones, supercomputers to artificial intelligence systems.

**1991**: Pierre-Gilles de Gennes was awarded the Nobel Prize in Physics for developing a generalized theory of phase transitions with applications to polymers.

**1994**: Stephen Z. D. Cheng and Frank W. Harris patent negative birefringent polyimide films for liquid crystal displays.

**1996**: Carbon nanotubes, a new form of carbon connected by covalent bonds, were discovered, exhibiting remarkable mechanical and electrical properties, influencing nanotechnology and materials science.

**2000**: Alan Heeger, Alan MacDiarmid, and Hideki Shirakawa were awarded the Nobel Prize in Chemistry for the discovery and development of conductive polymers.

**2005**: Robert Grubbs, Richard Schrock, and Yves Chauvin were awarded the Nobel Prize in Chemistry for their work on the development of the metathesis method in organic synthesis, important for polymer chemistry.

**2010**: The development of self-healing polymers begins, materials that can autonomously repair damage, extending the lifespan of polymer products.

**2014**: The first 3D-printed car, the Strati, is produced, showcasing the potential of additive manufacturing with polymers.

**2016**: Researchers develop the first recyclable thermoset plastics, addressing challenges in polymer sustainability and waste reduction.

**2018**: A new class of polymers called vitrimers was discovered, which combine the properties of thermoplastics and thermosets, allowing for reprocessing and recycling.

**2020**: Advancements in biodegradable polymers lead to increased use in packaging and disposable products, aiming to reduce environmental impact.

**2021**: High-performance polymers were developed for use in flexible electronics, enabling advancements in wearable technology.

**2023**: Artificial intelligence (AI) accelerates discovery and creation of new polymer systems.

To celebrate this century-long journey, PMSE organized a Centennial Symposium entitled “*PMSE Centennial: Celebration of Success and New Frontiers in Polymeric Materials Science and Engineering*” at the 2024 Spring ACS Meeting in New Orleans (Figure 1). This centennial symposium provided a rare platform to reflect on past scientific breakthroughs, highlight technological advancements, and explore new frontiers in the field of polymeric materials science and engineering. The symposium gathered 31 distinguished speakers from academia, industry, and national laboratories, each of whom shared their unique insights on the evolution and impact of polymeric materials. The event was structured into eight thematic areas: *Advanced Manufacturing, AI and Materials Discovery, Biomaterials, Electronic Materials, Energy, Entrepreneurship, Smart Materials, and Sustainability.* These themes encapsulate the broad spectrum of polymer science and engineering, underscoring both foundational contributions and emerging trends that will shape the field in the coming decades.

**Figure 1:**
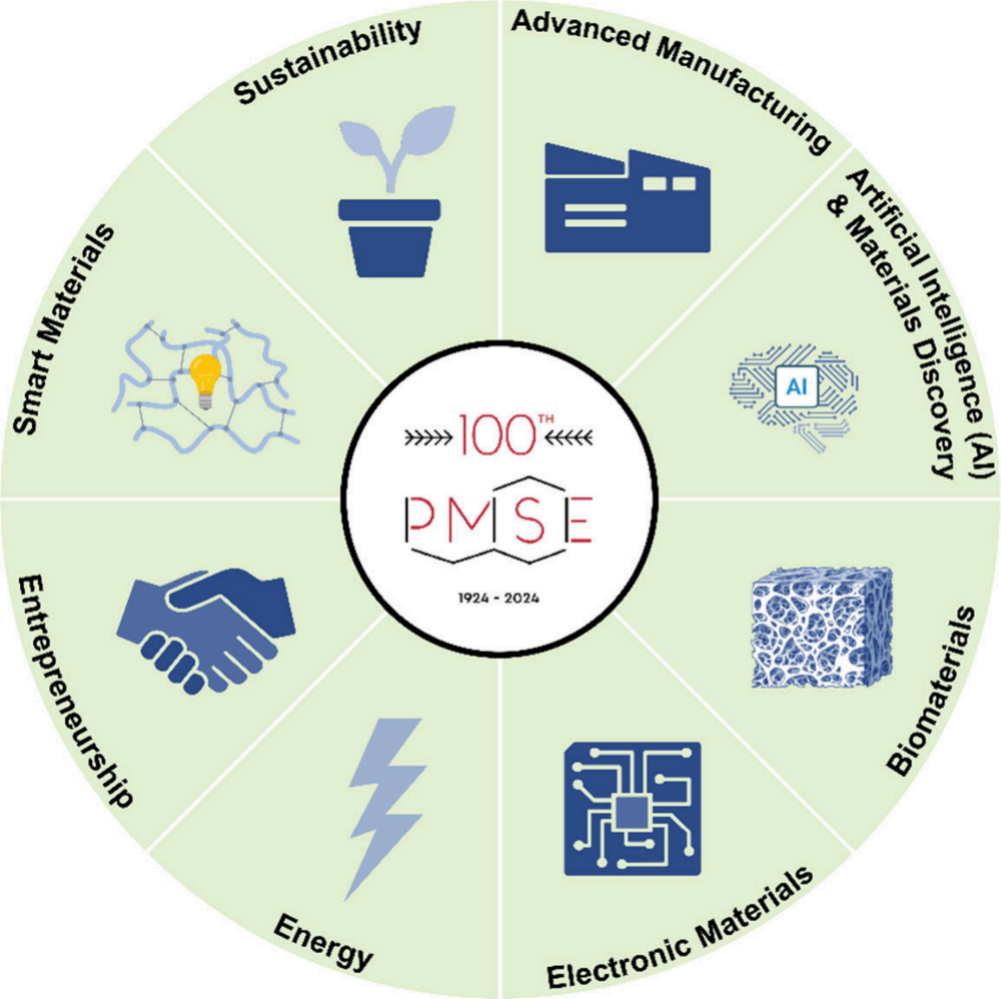
In 2024, the PMSE Division of ACS celebrated its 100th anniversary. To mark this milestone, the “PMSE Centennial: Celebration of Success and New Frontiers in Polymeric Materials Science and Engineering” symposium was held at the 2024 Spring ACS Meeting in New Orleans. The eight thematic areas highlighted foundational contributions and emerging trends.

A key aspect of the PMSE Centennial Symposium was its emphasis on interdisciplinary innovation and the role of polymeric materials in addressing pressing global challenges. Advances in *Advanced Manufacturing* highlighted the development of scalable and precise fabrication techniques, from additive manufacturing to nanofabrication, which have enabled new applications in electronics, healthcare, and sustainability. The *AI and Materials Discovery* session illustrated how computational modeling, machine learning, and automation are revolutionizing polymer design and accelerating the discovery of novel materials with tailored properties. In *Biomaterials*, speakers delved into the transformative impact of polymers in medical applications, ranging from smart hydrogels to bioinspired materials that enhance tissue engineering and drug delivery. *Electronic Materials* discussions focused on the role of polymers in next-generation flexible electronics, organic semiconductors, and biointegrated devices, reflecting the increasing convergence of polymer chemistry and electronic engineering. The *Energy* session examined the utility of polymers for energy storage and generation devices. The *Entrepreneurship* theme showcased the translation of polymer research into commercial success stories, emphasizing the importance of innovation, scalability, and industry-academic collaborations. The *Smart Materials* session examined the myriad of chemistries to provide on-demand properties for sensors and other applications. Finally, the *Sustainability* session underscored the urgent need for sustainable polymer solutions, including sustainable sourcing, recyclable or biodegradable polymers, and polymer-based energy storage technologies. The invited talks collectively painted a comprehensive picture of the field’s evolution while offering forward-looking perspectives on the future of polymeric materials. The following eight sections in this viewpoint expand on the symposium’s themes, summarizing key takeaways, identifying critical challenges, and exploring opportunities for continued advancements in polymer science. Through this reflection on the PMSE Centennial Symposium, we aim to celebrate the legacy of polymeric materials research while charting a path for the next century of innovation in this dynamic and burgeoning field.

## Advanced Manufacturing - Advincula

Soft matter and materials are key to novel technological opportunities in manufacturing, energy production, transport, and information technology.^2^ There has been a drive toward energy-efficient processes and sustainable materials in any manufacturing platform. The synergistic workflow of theory, computation, and physical experiments with functionality gain via optimization can accelerate materials discovery. In advanced manufacturing, the key is scalability with more complex designs and higher performance.^3^ While most manufacturing is still being done in batch or semibatch, there has been a lot of interest in continuous flow reactors and process engineering beyond vat-based reaction engineering. This brings more engineering from chemical plants to bench scale. Advanced manufacturing includes the use of AI and machine learning (AI/ML) with highly specialized automated hardware platforms that produce new and structured data for testing important key scientific hypotheses.^4^ Polymer materials have the advantage and richness of organic chemistry as part of the synthesis revolution that began and was catalyzed during World War II.^5^ The need for new rubber, plastics, nylon, and lightweight armor, followed by the space revolution with highly and thermally stable polymers and thermosets, has appended the synthesis of new materials. In addition, filler materials and composites can append their performance and processability without modifying the chemical structure or polymer microstructure with synergistic blending. Yet, processing and manufacturing have recently caught up, leading to new functions and applications. Traditional methods to form fibers, sheets, filaments, molding, vacuum-forming, and coatings have long been used in industry and are classified as formative manufacturing. Electrospinning, biaxial stretching, coaxial extrusion, layered melt polymer extrusion, and vacuum forming methods have been investigated using thermoplastics, elastomers, and thermosets in the last three decades. The term additive manufacturing (AM) has gained much attention due to using digital design with CNC or volumetric-driven extrusion, photopolymerization, sintering, and jettings.^6^ This results in more complex designs and shapes and simplification of the number of parts toward high efficiency and performance.^7–10^ Several examples and classes of these new manufacturing developments and applications include the following: (1) programmable liquid crystal elastomer structures via melt electro-writing, (2) elastomeric conducting inks for 3D-printed electronics, (3) shape memory materials from 3D printed nanocomposites, (4) polymers for electromagnetically driven actuator and remote robotics, (5) body-adapted fabrics and thermal management materials, (6) vitrimeric polymers and nanocomposites, (7) bioinspired hygro-responsive shape-changing deformations, and (8) high-performance polymers and geometries for extreme environments. The list is not exhaustive and is meant to be a small representation. For *de novo* sustainable materials (solid-state and soft-matter) to be scalable, high-performance, and recyclable (upcycle), they must be designed from their original inception.^11^ Engineering these systems with AI/ML-driven manufacturing may require multiple skills in algorithm development, design and fabrication of devices, sensors, mechatronics, and interfacing with more powerful computers for real-time learning and feedback loop experimentation. The expert polymer scientist will guide the “human-in-the-loop” experience to enable scientific discovery and process optimization toward auto-ML workflows that accelerate polymer materials discovery and manufacturing. In the coming years, we will see this revolution in how polymer synthesis and manufacturing will be done for many decades.

## AI and Materials Discovery - Jayaraman

AI, encompassing ML, automation, and high-throughput experimentation, has become increasingly prevalent in chemical sciences. AI presents tremendous value to chemical research by providing predictive models for complex phenomena, generating targets for new and improved materials, and automating and accelerating simulations/experiments. Over the past decade, polymer science and engineering has seen a major surge in research activity focused on developing, adopting, and using new and improved data-driven tools for predicting polymer properties and optimizing polymer synthesis without intensive trial-and-error experiments.^4,12–16^

One of the earliest perspectives on the topic of ‘AI for polymer science’ was published in 2017.^17^ This Perspective on ‘polymer informatics’ highlighted the opportunities and barriers for broader adoption of ML methods in polymer science. Unlike small molecule organic chemistry, inorganic chemistry, biochemistry, or colloidal chemistry, polymer science and engineering faces unique barriers for development of ML models and workflows. These barriers include challenges related to machine-readable representations or descriptors of polymers,^18,19^ lack of consistently labeled polymer databases,^4,17^ complexities arising from the diversity in morphologies (e.g., amorphous, crystalline, and semicrystalline) and structural length scales (e.g., monomer, chain, and domain) for automating structural characterization (see review articles^20,21^ ), and issues identifying optimal designs when processing alters the materials’ physical properties.^22–25^ These challenges have also created opportunities and inspired researchers to find solutions that advance the field of ‘AI for polymer science’.

Notable advancements in ‘AI for polymer science’ in recent years include reliable prediction of glass transition temperatures from monomer structures (citations in ref 26), AI-powered automated workflows accelerating polymer discovery (citations in ref 21), universal machine-readable descriptors for polymers (citations in ref 18), and ML workflows for fast and automated structural characterization (citations in ref 20). These advances have led to the development of promising new functional and sustainable polymers for various applications, including energy storage and biodegradable materials.^11,27,28^ As the community of polymer scientists continue to harness the transformative power of AI, the future of polymer science and engineering promises unprecedented innovations, driving us toward more sustainable and efficient macromolecular materials.

## Biomaterials – Cosgriff-Hernandez

Humankind has been using materials to augment or repair the body since ancient times. Historically, material selection was based on availability and focused on structural replacement of tissue lost due to disease or trauma. The field of biomaterials has undergone significant evolution, transitioning from inert materials to dynamic systems that actively respond to the biological environment.^29^ This shift from simple structural roles to active participation in biological processes has opened up new frontiers in medical devices, regenerative medicine, and drug delivery.

Advances in engineering from nanotechnology to 3D printing have enabled fabrication of biomaterials with increasing levels of sophistication and complex functions. Beyond chemical composition, increased control of topological, mechanical, and electrical cues has generated new classes of biomaterials for guiding biological responses.^29^ Dynamic materials that are able to transmit and respond to mechanical signals have enhanced the function of medical devices. For example, shape-memory materials that can change shape in response to temperature or other stimuli allow for minimally invasive deployment to reduce the need for extensive surgeries.^30^ The rise of computational modeling and design has also allowed for highly customized and optimized devices. By leveraging simulations, materials can be tailored for specific mechanical properties or interaction with tissues, reducing the trial-and-error approach in design and enhancing device performance.^31^

Regenerative medicine seeks to repair or regenerate damaged tissues and organs using biomaterials, often in conjunction with biological therapies. Modern biomaterial science has made great strides in matching the complexity of native tissues, which are frequently multifunctional and dynamic. These biomimetic materials can promote tissue regeneration by encouraging cell attachment, growth, and differentiation in ways that more closely resemble natural environments.^32^ In addition to the spatiotemporal control of cell-adhesion ligands, morphogens and other chemical cue presentation, hybrid biomaterials have been used to regulate the rate of matrix metalloproteinase-mediated degradation and cellular invasion.^33,34^ More recently, biomaterials with the ability to sense and respond to mechanical stimuli (e.g., stress relaxation, dynamic stiffening) have been used to guide cell behavior.^35^ A growing area of regenerative medicine is the manipulation of the immune system.^36^ Biomaterials are being engineered to release immunomodulatory cues, recruit regulatory immune cells, or shift macrophage polarization to enhance regeneration, promote vascularization, and prevent fibrosis.

Drug delivery systems have benefited from polymer and lipid-based materials for controlled release of therapeutic agents to maximize the desired therapeutic effect and reduce the toxicity to the patient. In addition to enhancing oral and injectable drug delivery, new routes of administration for drug delivery have been achieved including pulmonary, transdermal, ocular, and nasal routes.^37^ In addition, targeting modalities including aptamers and antibodies have been used to deliver drugs to specific tissues or cells to improve the precision of drug delivery, minimizing side effects and enhancing therapeutic efficacy.^38^ With the rise of RNA therapies, lipid nanoparticles and other biomaterial carriers have become integral to mitigate immunogenicity, increase stability, promote cellular uptake, and improve therapeutic efficacy.^39^ A key advance in drug delivery systems is the development of smart biomaterials that respond to chemical, biological, or physical stimuli (e.g., temperature, pH, light, electromagnetic fields).^40^ These materials can release drugs on demand or with a physiological trigger, enhancing the specificity and control over therapeutic interventions. Finally, theranostic biomaterials combine both therapeutic and diagnostic functions, allowing simultaneous treatment and monitoring of diseases. By integrating diagnostic agents with therapeutic ones, clinicians can track the progress of treatment in real time and adjust interventions accordingly.^41^ Collectively, these advancements reflect the ongoing trend of making biomaterials more sophisticated, responsive, and integrated with biological systems, transforming the way we approach patient care and therapy.

## Electronic Materials - Reichmanis

Harkening back to 1967 and the quote from the movie “*The Graduate*” about the future of plastics,^42^ polymers have played and will continue to play important roles in almost every aspect of our lives. From the perspective of electronics technologies, they were used early on as insulators for electrical cables; and notable discoveries made by Lincoln Hawkins, Vincent Lanza, and Field H. (Stretch) Winslow in the 1950s led to the development of additives for stabilizing polyethylene allowing its use as a coating for communications cables.^43,44^ Subsequently, advancements in photopolymer chemistries resulted in the development of advanced highly sensitive materials and processes that led to today’s microelectronic photoresists.^45^ Without such photopolymer systems, there would be no computers, microprocessors, or high-capacity data storage systems and, consequently, no microelectronics revolution.

The predominant technology used to fabricate state-of-the-art devices continues to be optical lithography. Since the late twentieth century, photopolymer systems that respond to 248 and 193 nm excimer laser light sources were designed and introduced into device manufacturing lines allowing sub-20 nm scale resolution of features that represent circuit elements.^45–47^ As state-of-the-art device technology continues to demand higher resolution imaging, extreme ultraviolet (EUV) sources are emerging as the next lithographic technology frontier. The shorter wavelength of EUV enables definition of smaller critical dimensions; however, EUV presents new research challenges, particularly surrounding the design of photoresist chemistry and processes that will allow imaging circuit features that may be no more than a few nm in size.^48,49^

Within the realm of electronic materials, polymeric materials can also be designed to serve as electronic conductors and semiconductors.^50^ Silicon-based microelectronics technology has enabled the unprecedented advancements in a wide range of technologies and similarly today, there are a myriad of emerging applications and opportunities surrounding (opto)­electronic polymer-based devices. While polymers are not likely to reach the levels of performance achieved by their silicon counterparts, low-cost polymer-based flexible electronics provides opportunities to revolutionize how we use devices in applications ranging from energy storage to sensors for environmental and health monitoring.^51,52^ Underlying the successful design, development, and implementation of these emerging materials chemistries is identification of a set of ‘design rules’ derived from fundamental structure–property relationships, coupled with the development of process-structure–property relationships that govern molecular organization.^53,54^ Device performance depends critically on surfaces, interfaces, and active material assembly/alignment at many length scales, and these considerations will undoubtedly impact the design and development of advanced new (opto)­electronic materials technologies for consumer electronics, biomedical, environmental and communications applications.

## Energy - Lutkenhaus

Polymers are required in nearly every energy storage and generation application, from the membranes used in fuel cells to the binders in lithium-ion battery electrodes. However, many energy storage and generation devices require strategic materials that are highly sensitive to supply and demand. Examples include cobalt and nickel in lithium-ion battery electrodes and platinum in fuel cell electrodes. Further, other energy storage and generation platforms utilize environmentally sensitive elements, such as lead in perovskite solar cells or vanadium in reduction–oxidation (redox) flow batteries. Replacing these problematic inorganic materials with polymeric ones offers a strategy toward increasing materials circularity, especially if those polymeric materials are sustainably sourced.

In batteries, polymers are essential components as electrode binders (e.g., polyvinylidene fluoride) and as separators (e.g., polypropylene, polyethylene), but mostly as passive additives. However, polymers can act as active components, such as solid polymer electrolytes or redox-active polymer electrodes.^55^ Research in polymer electrolytes has long centered on controlling the segmental dynamics of polymer chains for ion conduction, but emerging work shows that superionic conductivity can be achieved in polyzwitterions.^56^ This approach centers on decoupling ion conduction from polymer chain dynamics. Decreasing the energy barriers to ion transport^57^ is a key goal. For electrodes, redox-active polymers are emerging in interest for their fast-charging capabilities and independence from strategic metals.^58^ The main challenges are to increase the active material loading and to increase stability and activity. It is also of essential importance to understand how electrons and ions move because these redox-active polymers are also mixed ion conductors.^59^ Emerging work also examines redox-active polymer recycling,^60^ on-demand degradation,^61^ and biomass sourcing.^62^

Perovskite and silicon solar cells show exceptional efficiencies, but bulk heterojunction polymer solar cells continue to improve.^63^ The state-of-the-art approach in polymer solar cells centers on bulk heterojunction technology, which requires careful manipulation of interfacial charge separation and transport. Current research is intensely focused on crystallization optimization to control self-regulation at the heterojunction interface. Adding small molecules as crystallization agents is one promising approach,^64^ as is side-chain engineering to control molecular packing.^65^

In fuel cells, intense materials innovation is centered on improving the ion-conducting membrane. With new concerns over perfluoroalkyl substances and fluorine-containing materials, alternatives to fluorine-containing Nafion are needed. Copolymers are one promising approach, in which ion conduction, mechanical robustness, water management, and chemical stability must be balanced.^66,67^ Further, anion-exchange membranes are key components in alkaline fuel cells, but their stability and conductivity need improvement. The most challenging aspect is maintaining stability of the membrane in the extremely alkaline environment at elevated temperatures. Nucleophilic attack of the cationic group on the polymer is the main source of instability, but this process can be mitigated by careful tuning of the cation’s chemistry and how it is linked to the main chain.^68,69^

The key theme in polymers for energy storage and generation applications is balancing stability with performance. Redox-active polymers in batteries should have high energy densities and long-cycle lives; similarly, solid polymer electrolytes should conduct ions without decay. Organic bulk heterojunction solar cells need vast improvements in efficiency and improved stability to oxygen and moisture. Ion-exchange membranes for fuel cells require high conductivity and stability in extremely acidic or alkaline conditions. Therefore, many opportunities are available for polymer scientists and engineers to design the next generation of polymer materials to create a greener and more sustainable energy storage landscape.

## Entrepreneurship - Priestley

The journey of polymer entrepreneurship, from Leo Baekeland’s groundbreaking commercialization of Bakelite to today’s innovative polymer startups, represents a remarkable century of translating scientific discovery into societal impact.^70^ Baekeland, who eventually became a professor at Columbia University, founded the General Bakelite Company to commercialize his discovery. While historians have marked the discovery of Bakelite as the beginning of the plastics age,^71^ we may also consider it the beginning of a dynamic intersection between academic research and polymer entrepreneurship as a means of transforming laboratory discoveries into commercial solutions that improve the quality of life.

As revealed in the PMSE Centennial section “Entrepreneurship,” the model of academic spinouts has evolved into a sophisticated ecosystem where polymer scientists increasingly approach research with an entrepreneurial mindset, supported by university technology transfer offices, incubators, and a growing network of investors who understand the unique challenges and opportunities in materials innovation.^72^ From biodegradable polymers for drug delivery and medical devices, to conductive polymers for electronic applications, to sustainable plastics to reduce the carbon footprint, to polymer formulations for advanced personal care, to polymer processing technologies for miniaturization, these technologies have all benefited from rigorous, long-term academic research before translation into solutions that touch nearly every aspect of modern life. Indeed, university startups are built on the foundation of basic research, as the genesis of many can be traced back to peer-reviewed publications. The connection between basic research and entrepreneurship has been recognized as a critical link of innovation by funding agencies, including the National Science Foundation (NSF). While the NSF has historically supported fundamental research, it now comprises the Directorate for Technology, Innovation, and Partnerships, which aims to accelerate technologies and foster economic growth.^73^

Knauer and colleagues, in a viewpoint, provided the most comprehensive overview and “how-to-guide” for aspiring polymer entrepreneurs.^74^ They framed the startup journey as analogous to synthesizing a new molecule (i.e., having a starting point but requiring adaptability and creativity to reach the desired outcome). The article outlined several critical aspects of polymer science entrepreneurship, including team building, funding strategies, and unique technical challenges. The article also focused on the evolving ecosystem supporting polymer entrepreneurship, particularly within academic institutions, including the NSF I-Corps program,^75^ which provides immersive entrepreneurial training to scientists and engineers. We point the interested reader to the article and wish them good luck in their potential entrepreneurial journey, should they choose to pursue one.

## Smart Materials - Voit

Smart polymeric materials have a very rapid development in the last 30 years with impressive examples of applications, e.g., in biomedicine, flexible electronics, sensorics, and robotics. Beside developing the tools for the introduction of designed, specific functions in a macromolecule, significant progress has been made with regard to device integration, allowing development of new smart technologies and devices, and lately also considering the aspect of sustainability. Smart materials comprise various types of polymeric materials that show a specific response upon any chemical or physical trigger, which might be manifested as changes in ion and electrical conductivity, in optical behavior and color, in shape and size, in mechanics, etc. New efficient reaction schemes, a deeper understanding of the underlying physical principles to achieve the desired responses, and especially the interdisciplinary interactions derived from computer science, medicine, and electrical engineering have broadened significantly the material design and application scope, and one can imagine, that tools of machine learning and artificial intelligence will further boost the development of such complex materials. This perspective will briefly highlight three different areas in *Smart Polymeric Materials*:

### Smart Soft Matter and Adaptive Materials

The sense–assess–respond feedback loop is the guiding design construct for many smart polymer materials. The terms “smart”, and more generally, “adaptive” materials have been applied to material systems that possess all or portions of this feedback loop.^76^ Such adaptive polymers, predominantly hydrogels, can react to their environment by a specific actuating response, such as changing volume, size, mechanics, or shape. Beside a rather simple unidirectional swelling/deswelling in a hydrogel by pH or temperature change, often used for drug release, much more complex interactions can be realized in smart soft matter, allowing processing via multiple chemical as well as physical information, often in a synergistic way.^77–79^ Recent examples cover, including directed motion of microrobots potentially through chemotaxis in a biological environment,^80–82^ the automated control of complex multistep reactions in a microfluidic chip up to “chemical computing”,^83,84^ or the simulation and control of biological pathways,^85^ potentially useful in in organoid cultures. Special attention is given to producing autonomous systems to acquire the energy needed for action directly derived from the biological system^80^ or in synthetic dissipative systems.^86^ Extending these materials concepts of feedback and distributed energy to systems-level challenges, such as in soft robotic platforms, prompts the consideration of the physical integration or “embodiment” of information^87,88^ and energy propagation in the system.^89^ Leveraging sense–assess–responds feedback dynamics for information processing and control is a promising direction on the path toward autonomous, adaptive materials.

### Flexible Electronics

Smart flexible electronic and wearable devices, which combine information collection (sense), information processing (assess), and output action (respond), have the additional criteria of robust electronic properties, biocompatibility, and low-cost readily accessible material precursors.^90^ Organic and polymeric semiconductors are the basis for that, but maintaining the electronic properties in combination with elastic (soft) behavior is a big challenge. Much progress had been made, for example with regard to control over the morphology and ordered structure, and integration of semiconducting moieties into hydrogels and blends to enable tough and stretchable semiconductor materials suited for integrated circuits.^91,92^ These advance have made bioelectronics a very hot topic for innovations in medicine providing effective electronic skins^93^ and smart brain implants,^94^ and bridging the gap between electronics and the biosystem via flexible, organic semiconductor materials.

### Dynamic Bonding

Smart materials have to be of a dynamic nature, meaning properties can vary with time and on specific triggers. Introducing dynamic bonding, noncovalent or covalent one, is a highly important aspect of this design, which brings not only the possibility of self-healing and recycling, but also embodies function into materials.^95,96^ The introduction of the material classes of “vitrimers” ^97,98^ and “covalent adaptable networks”^99^ has significantly influenced the polymer community in the past few years, addressing important sustainability aspects in the development of more recyclable, functional materials, especially engineering polymers and thermosets. Increase of lifetime by a dynamic, reformable bond is highly important for elastomers and thermosets, but offers also a platform to induce flexibility into polymeric semiconductors. Recycling on demand, where a bond is cleaved in a defined way in the presence of a trigger (pH, catalyst, light, heat) can also be used for introducing sensing function. Thus, mechanical properties needed for specific applications such as bulk materials can be combined with desired functionality as well as options for recycling, reuse, and in general, reduction of plastic waste.

Recent developments have shown huge progress in the chemistry of dynamic bonds (e.g., very fast and efficient bonding by triazolinediones [chemistry),^100^ or use of photoinitiated thiol–ene chemistry,^101,102^ enabling their application in technically relevant processes and materials.^103^ Still significant challenges remain to substitute more and more of the conventional thermosets and thermoplastic materials with a more sustainable solution based on dynamic bonding.

## Sustainability - Epps

Lignin is an ideal natural source of aromatic building blocks, and lignin deconstruction can potentially provide valuable, aromatic, additives, monomers, solvents, and platform chemicals.^104,105^ While there are well-researched pathways for the valorization of biomass-derived cellulose and hemicellulose into fuels, solvents, chemicals and materials, lignin is a complex aromatic polymer whose conversion is challenging due to its inherent recalcitrance.^106^ Presently, lignin is separated from biomass at ∼100 Mt/y through pulping or biorefining processes,^107^ but most isolated lignins have broad a molecular weight distribution, dark color, strong odor, and limited reactivity.^108^ There is also enormous variability in composition, chemical structure, cost, and environmental footprint of isolated lignins due to differences across feedstocks (source and location) and pulping/refining techniques. Moreover, the deconstruction of lignin generates a complex mixture of disparate compounds (monophenols, dimers, and oligomers).^106,107,109,110^

While some efforts have focused on the direct use of chemicals/monomers from raw biomass deconstruction,^111^ the majority use lignin model compounds that have been substituted as mimics to formulate of new products or employ real lignin for very low-value applications (e.g., concrete and tire fillers, dust suppressants). Essentially, a sizable gap exists between deriving well-defined chemicals from lignin-rich biomass and directly utilizing these chemicals for higher-performance polymeric materials and valuable platform chemicals.^106^

Four research aspects can further accelerate the translation of lignin-derived molecules from fundamental research to widespread application: (1) the development of robust source-structure–property-processing relationships that span from lignin mimics to real mixtures;^112^ (2) the demonstration of lignin-derived, performance-advantaged materials in high-value applications;^113^ (3) the investigation of process intensification routes to improve the cost- and energy-efficiency of lignin deconstruction and product purification routes;^106,114,115^ and (4) the integration of more holistic life-cycle assessment (LCA) and technoeconomic analysis (TEA) approaches that account for all aspects of lignin valorization from harvest to end of life/reuse.^106,116^ In the first case, the source of the lignin has a significant impact on the structure/composition of the phenolics obtained from deconstruction; these outputs, in turn, effect the ultimate materials properties (thermal, chemical, mechanical) that dictate the processing conditions and methods needed to generate desirable products. In the second case, while it is useful to suggest that biobased materials may be useful for a given application, a tangible demonstration that highlights the potential performance-advantages of a lignin-derived output provides a useful framework that could facilitate industrial adoption.^117^ In the third case, lignin is considered a renewable and sustainable resource,^108,112,118^ but in many instances, the deconstruction of the feedstock and the isolation of the desired products involves processes that consume large quantities of energy, harsh chemicals, waste generation, and greenhouse gas emissions.^115,119^ While the overall cradle-to-materials umbrella may still be preferable to approaches that utilize nonrenewable feedstocks,^116^ there is substantial room to reduce costs and environmental impacts (e.g., through approaches that combine reaction and separations^114^) or leverage membrane-based purification vs distillation.^119,120^ In the fourth case, reports that focus on the fundamental properties and application of biobased materials present the benefits of renewable and potentially sustainable feedstocks without detailed consideration of the feedstock procurement process,^121^ thus omitting information necessary to assess needs, such as centralized vs distributed supply chain management and variability in feedstocks,^122^ along with feedstock quantities available for realistic sourcing. Finally, it is worth noting that all biobased materials are not biodegradable or inherently reprocessable;^123^ hence, it is necessary to consider recycling approaches similar in function to those in development for common polymers and plastics.^121^ Overall, much progress has been made, and significant opportunities exist to unlock the use of biomass (and lignin) as a high-value feedstock.
